# *Bacillus halotolerans* SW207 alleviates enterotoxigenic *Escherichia coli*-induced inflammatory responses in weaned piglets by modulating the intestinal epithelial barrier, the TLR4/MyD88/NF-κB pathway, and intestinal microbiota

**DOI:** 10.1128/spectrum.03988-23

**Published:** 2024-03-07

**Authors:** Minghan Li, Dongyu Zhao, Jialin Guo, Tianxu Pan, Tianming Niu, Yanqi Jiang, Chunwei Shi, Haibin Huang, Nan Wang, Di Zhang, Shumin Zhang, Chunfeng Wang, Guilian Yang

**Affiliations:** 1 College of Veterinary Medicine, Jilin Agricultural University, Changchun, China; 2 Jilin Provincial Engineering Research Center of Animal Probiotics, Jilin Provincial Key Laboratory of Animal Microecology and Healthy Breeding, Jilin Agricultural University, Changchun, China; 3 Engineering Research Center of Microecological Vaccines (Drugs) for Major Animal Diseases, Ministry of Education, Jilin Agricultural University, Changchun, China; National Institutes of Health, New York, USA

**Keywords:** enterotoxigenic *Escherichia coli*, barrier function, host-microbe interactions, oxidative stress

## Abstract

Enterotoxigenic *Escherichia coli* (ETEC) is one of the major pathogens contributing to piglet diarrhea, with significant implications for both piglet health and the economic aspects of the livestock industry. SW207 is an isolate of *Bacillus halotolerans* isolated from the cold- and disease-resistant Leixiang pigs in Northeastern China. We have discovered that SW207 can survive in the pig’s gastrointestinal fluid and under conditions of high bile salt concentration, displaying potent antagonistic activity against ETEC. In this study, we established a weaned piglet diarrhea model infected with ETEC to investigate the role of SW207 in preventing diarrhea and improving intestinal health. Results indicate that SW207 upregulates the expression of tight junction proteins, including claudin-1, occludin, and zonula occludens-1, at both the transcriptional and translational levels. Furthermore, SW207 reduces serum endotoxin, D-lactic acid, and various oxidative stress markers while enhancing piglet mechanical barrier function. In terms of immune barrier, SW207 suppressed the activation of the TLR4/MyD88/NF-κB pathway, reducing the expression of various inflammatory factors and upregulating the expression of small intestine mucosal sIgA. Concerning the biological barrier, SW207 significantly reduces the content of *E. coli* in the intestines and promotes the abundance of beneficial bacteria, thereby mitigating the microbiota imbalance caused by ETEC. In summary, SW207 has the potential to prevent weaned piglet diarrhea caused by ETEC, alleviate intestinal inflammation and epithelial damage, and facilitate potential beneficial changes in the intestinal microbiota. This contributes to elucidating the potential mechanisms of host-microbe interactions in preventing pathogen infections.

**IMPORTANCE:**

Enterotoxigenic *Escherichia coli* (ETEC) has consistently been one of the significant pathogens causing mortality in weaned piglets in pig farming. The industry has traditionally relied on antibiotic administration to control ETEC-induced diarrhea. However, the overuse of antibiotics has led to the emergence of drug-resistant zoonotic bacterial pathogens, posing a threat to public health. Therefore, there is an urgent need to identify alternatives to control pathogens and reduce antibiotic usage. In this study, we assessed the protective effect of a novel probiotic in a weaned piglet model infected with ETEC and analyzed its mechanisms both *in vivo* and *in vitro*. The study results provide theoretical support and reference for implementing interventions in the gut microbiota to alleviate early weaned piglet diarrhea and improve intestinal health.

## INTRODUCTION

Enterotoxigenic *Escherichia coli* (ETEC) is an intestinal pathogen that elicits diarrhea in both human and livestock, with a particular predilection for young individuals (1, 2). Human exposure to ETEC typically occurs via ingestion of food and water that has been contaminated, resulting in acute watery diarrhea, headache, vomiting, and other severe health risks (3, 4). In the swine industry worldwide, ETEC is a primary factor contributing to post-weaning diarrhea in pigs, resulting in significant economic losses (5). In 2022, China enacted regulations prohibiting the use of certain critical antibiotics in animal production and concurrently strengthened antibiotic oversight. The new substitutes of antibiotics are needed as feed additives to improve animal health, prevent the onset of diseases, and improve production performance.

Studies have indicated that probiotics demonstrate efficacy in enhancing gastrointestinal health (6–8) and regulating immunity (9) as well as inhibiting the growth of pathogens (10), providing a possible strategy for preventing and treating some foodborne intestinal disorders. Intestinal health is essential in terms of metabolism and nutrition absorption (11) while also serving as an important protective barrier against external pathogen invasion (11). The function of intestinal barrier and the stability of microflora are inseparable from the integrity and stability of intestinal epithelial barrier. Intestinal epithelial tight junctions are predominantly constituted by transmembrane and structural junction proteins (12). The mucus layer serves as the chemical defense barrier of the intestinal tract, working alongside the intestinal immune barrier to safeguard the intestinal epithelium against pathogen invasions (13). Studies have elucidated that ETEC induced an exaggerated state of oxidative stress in piglets, increased intestinal epithelial permeability, and damaged intestinal barrier function (14). However, some probiotics have the potential to assist hosts in resisting damage caused by chemicals or pathogens while maintaining intestinal barrier function and reducing damage (5). *Bacillus* is one of the most important species of probiotics, characterized by the production of spores, rapid reproduction, strong stress resistance, and strong colonization ability (15, 16). Studies have found that *B. amylolyticus* (9) demonstrates the potential to enhance piglets’ growth performance, immune level, and gut health.

The activation of the TLR4/MyD88/NF-κB signaling pathway within the intestinal tract leads to the secretion of numerous inflammatory factors, triggering a broad inflammatory response. This disrupts the structural integrity of the intestinal epithelium, thereby compromising both the mechanical and immune barrier functions of the gut. *B. licheniformis* (14) has been found to play a pivotal role in modulating the innate immune system of infected individuals. It reduces the production of TNF-α, IL-8, and IL-6 by inhibiting the NF-κB signaling pathway, thus mitigating ETEC-induced damage to IPEC-J2 cells.

Despite numerous studies clarifying the significant roles of various *Bacillus* in regulating health, there remains limited research on the impact of *B. halotolerans* on intestinal barrier and inflammation, both *in vitro* and *in vivo*. In this study, we isolated a versatile antibacterial strain, *B. halotolerans* SW207. We intend to utilize a weaned piglet diarrhea model infected with ETEC to primarily investigate the protective role of SW207 in ETEC-induced intestinal inflammation and ecological disruption from the perspectives of intestinal immunity, biological, mechanical barrier, and oxidative stress. Our findings illuminate the significant potential and molecular mechanisms of SW207 in preventing ETEC infections.

## MATERIALS AND METHODS

### Microbial strains and growth conditions

The ETEC reference strain C83902 (O8: H19: F4ac^+^ LT^+^ STa^+^ STb^+^) was a gift from Prof. Guoqiang Zhu, Yangzhou University, China. *Staphylococcus aureus* (USA300), *Salmonella enterica* serovar Typhimurium (CICC 21484), and *Actinobacillus pleuropneumoniae* (APP, ATCC 27090) were collected from the China Center of Industrial Culture Collection. Pathogens were grown in Trypticase Soy Broth (TSB) medium at 37°C for 12 h; the experiments proceeded using Optical density (OD) values ranging from approximately 0.6 to 0.8. The strain of *B. halotolerans* SW207 was isolated from a healthy Lei-Xiang pig’s cecum (Qipan Ecological farm, Jilin Province, China) and has been preserved in the China Center for Type Culture Collection (no. M20221856, Wuhan, China). The SW207 strain was grown in TSB medium at 37°C for 16 h; the experiments proceeded using OD values ranging from approximately 0.8 to 1.0.

### Morphological characteristics of *B. halotolerans* SW207

For observation in the scanning electron microscope (SU8100, Hitachi, Japan), the bacteria precipitation was fixed by electron microscopy fixative; the tissue blocks were transferred to a solution of 1% OsO_4_ and incubated for 2 h. After dehydration, the sample is put into a critical point dryer (K850, Quorum Technologies, UK) for drying. The specimens were affixed to metallic stubs using carbon stickers and subsequently sputter coated with a layer of gold for a duration of 30 seconds. Observe and take images with the scanning electron microscope.

For observation in the transmission electron microscopy (TEM, HT7800, Hitachi, Japan), the bacterial precipitation was pre-embedded in agar and subsequently subjected to post-fixation, room temperature dehydration, osmotic embedding, and polymerization for a duration of 48 h and sliced (60–80 nm) with an ultra-thin microtome (Leica UC7, Leica, Germany). The sections were then subjected to uranium-lead double staining (with a 2% uranyl acetate saturated aqueous solution and lead citrate, each staining process taking 15 min), followed by overnight drying at room temperature. Observe and take images with TEM.

### Survivability in gastrointestinal tract (GIT environment of *B. halotolerans* SW207

The tolerance *in vitro* of strain was assessed using a methodology that was described previously (17) with slight modifications. Swine GIT fluids were collected following previously described methodologies (18). Then the obtained supernatant was sterilized using a 0.22 μm filter and used for the subsequent experiments. The strain was introduced into the TSB broth supplemented with varying concentrations of swine bile salts (0.1%, 0.2%, 0.3%, 0.4%, and 0.5%), hydrochloric acid at different pH levels (2, 3, 4, 5, and 6), and swine gastric fluids (pH 3.8) and small intestinal fluids (pH 6.0). After 3 h of co-cultivation, the survival rate was assessed using a methodology that was described previously (10).

### Evaluation of antibacterial activity of *B. halotolerans* SW207

#### Cell-free supernatant (CFS) antibacterial test for SW207

The inhibitory activity of the CFS from SW207 was assessed using previously described procedures with slight modifications (19). The pathogenic indicator bacteria included ETEC, *S. aureus*, *S. enterica* serovar Typhimurium, and APP. In the bacteriostatic test, the working concentration of all pathogen indicator bacteria was 1 × 10^8^ CFU/mL. In three separate Oxford cups, 200-µL CFS of SW207, sterile water, and 200-µL penicillin (0.1 mg/mL) solution were added, respectively.

#### Agar spot antibacterial test for SW207

The contact-dependent inhibitory activity of agar spot from SW207 was assessed using previously described procedures with slight modifications (20). Two microliters of SW207, 2-µL sterile water, and 5-µL 1 mg/mL penicillin were dropped into the solid medium, respectively.

The presence of antimicrobial activity was tested by the absence of any visible growth inhibition surrounding the cups or colony. The diameter of the inhibition zone was tested in millimeters, starting from the outer edge of the cup or colony. The experiment was performed in triplicate.

### Animals

**Fig 1:**
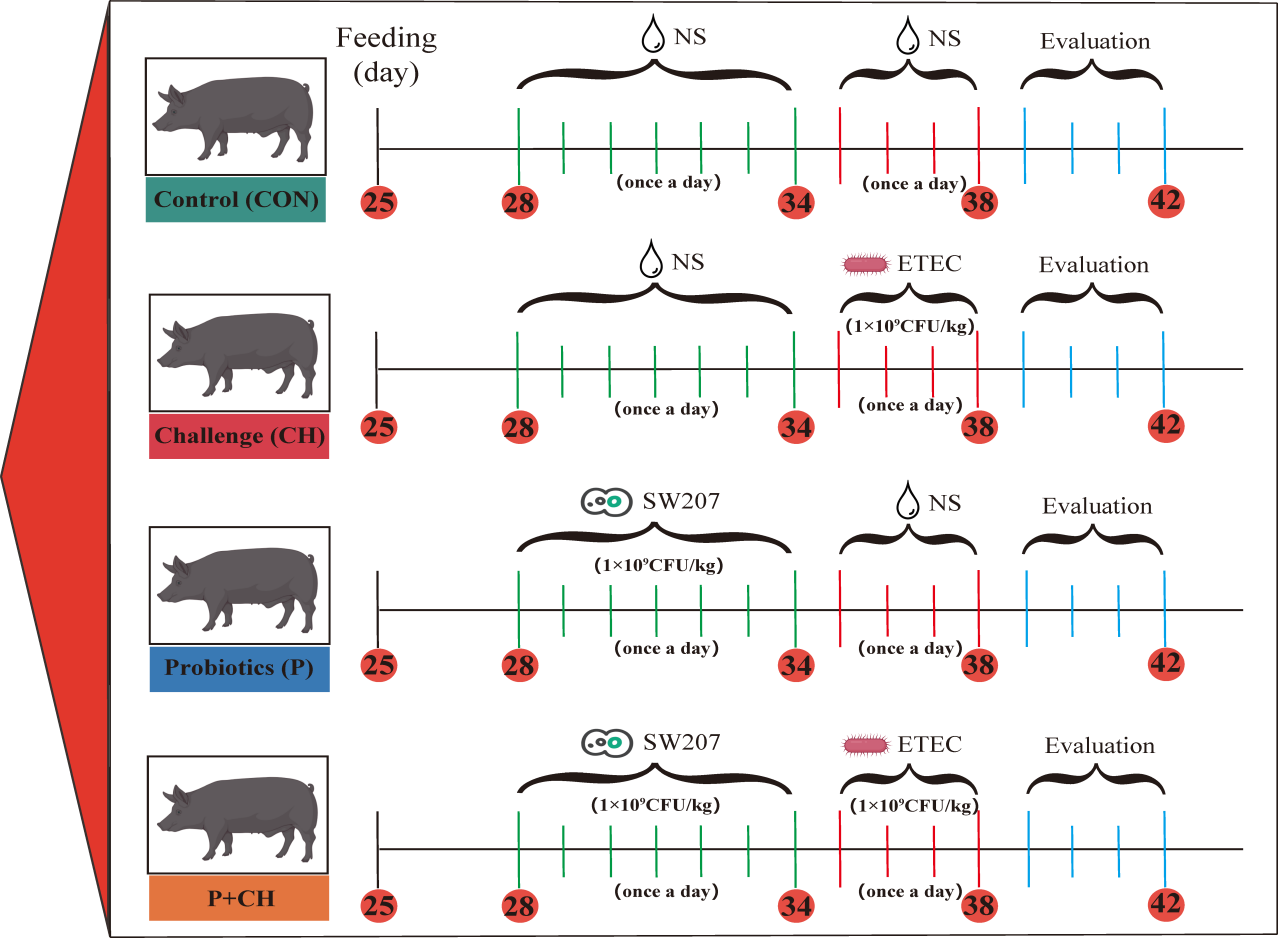
Animal experiment design. ETEC, enterotoxigenic *Escherichia coli*; SW207, *Bacillus halotolerans* SW207; NS, normal saline.

All the procedures were approved by the Institutional Animal Care and Use Committee at Jilin Agriculture University (2023 06 12 001). A total of 12 litters of Songliao Black weaned piglets (six to eight piglets in a litter) were selected from a commercial pig farm in Gong Zhuling, Jilin, China, based on the piglet birth weight, sow breed, date of birth, and litter size. The experimental design and animal treatment scheme are depicted in Fig. 1. Four treatments were randomly assigned to the weaned piglets, namely, the CON group (no treatment control, three litters), the CH group (ETEC infection, three litters), the P group (SW207, three litters), and the P + CH group (SW207 prevention of ETEC infection, three litters). Following a 3-day adaptation period, piglets in the CON group were orally gavaged with Phosphate-buffered saline (PBS); those in the P group were orally gavaged with resuspension of SW207; those in the CH group were orally administered with a resuspension of ETEC (1 × 10^9^ CFU/kg) (21); and those in the P + CH group were orally gavaged with a resuspension of SW207 and ETEC, ensuring that the amount of liquid used in oral gavaged is consistent. The experimental plan will strictly adhere to the time points outlined in Fig. 1. On day 43, a piglet with a body weight close to the average weight of the litter was selected from each litter and subsequently euthanized for sampling purposes. Serum samples were collected via blood sampling from the jugular vein prior to the euthanasia procedure. Intestinal samples were obtained from piglets at a location proximal to the midpoint of the intestine, encompassing the duodenum, jejunum, and ileum. Segments measuring approximately 2–3 cm in length were preserved in 4% paraformaldehyde for subsequent analysis of intestinal morphology. The mucosa, obtained from the intestine, was subjected to a thorough wash with PBS and then promptly frozen at −80°C. The contents of cecum were collected for sequencing of the 16S rDNA.

### Diarrhea incidence analysis

Fecal consistency was evaluated five times daily from day 39–42 and assigned scores according to established methodologies reported in previous studies (22, 23).

### Determination of *E. coli* load in cecum contents

In order to determine the load of *E. coli* in cecal contents, the cecal contents were homogenized and diluted by gradient (10^2^–10^6^). The diluted content homogenate (100 µL) was inoculated into eosin methylene blue (EMB) agar plate and incubated overnight at 37°C. The bacterial load in cecal contents was determined by counting the number of different concentrations of bacteria on EMB plates (colony-forming unit per gram).

### Serum parameter analysis

The blood samples were subjected to centrifugation at 2,500 rpm for 15 minutes to separate the serum. The serum levels of endotoxin and D-lactic acid were determined following the instructions provided in the respective kits (Nanjing Jiancheng, China). Meanwhile, serum parameters including IL-6, IL-1β, IL-8, TNF-a, IL-10, and sIgA were determined using Enzyme linked immunosorbent assay (ELISA) kits (Jiangsu Meimian Industrial Co., China). In addition, serum Myeloperoxidase (MPO), Total Antioxidant Capacity (T-AOC), Glutathione Peroxidase (GSH-PX), Glutathione S-transferase (GSH-ST), Total Superoxide Dismutase (T-SOD), Catalase (CAT), and Malondialdehyde (MDA) were determined utilizing commercially available assay kits (Nanjing Jiancheng, China). The experimental procedures strictly adhered to the instructions provided by the manufacturers.

### Real-Time Quantitative Reverse Transcription PCR (qRT-PCR)

Total RNA was extracted from ileum homogenates and reverse transcribed into cDNA following established procedures (24). qRT-PCR analysis was carried out using the QuantStudio 5 (ABI, USA) instrument. The 2-ΔΔCt method (25) was employed to calculate the relative mRNA levels of two distinct gene fragments normalized to β-actin. The primer sequences used in this study are listed in Table S1.

### Western blotting

Western blot analysis was following the protocol used in our laboratory (26). The quantification of protein bands was performed using the Amersham Imager 680 system (General Electric Company, USA). The density of the specific bands was measured utilizing ImageJ software. The following antibodies were used for this research: NF-κB antibody (ab32536, Abcam Plc, UK), p-NF-κB antibody (ab86299), zonula occludens-1 (ZO-1) antibody (AF5145, Affinity Biosciences, USA), claudin-1 antibody (AF0127), occludin antibody (AF4605), TLR4 antibody (AF7017), MyD88 antibody (AF5195), and beta-actin monoclonal antibody (AF7018).

### Histopathology

Intestinal morphology was examined using hematoxylin and eosin (H&E) staining, following the established protocol in our laboratory (27). Digital images of the intestinal morphology were captured using a microscope (Leica, Germany) at magnifications of 40× and 100×. For each sample, five fields were randomly chosen to measure the villus height and crypt depth.

Intestinal goblet cell numbers were assessed using periodic acid Schiff (PAS) staining, following the established protocol in our laboratory (27). Goblet cells within the intestinal region were subjected to staining using a magenta dye. The intestinal morphology images were captured using a light microscope at magnifications of 40× and 100×. Five villus-crypt units were randomly chosen for the enumeration of goblet cells.

### Microbial analysis of gut microorganisms

Total genomic DNA from the samples was extracted using the Cetyl Trimethyl Ammonium Bromide/Sodium Dodecyl Sulfate (CTAB/SDS ) method. The PCR product mixture was purified using the QIAquick Gel Extraction Kit. Sequencing libraries were prepared using the SMRTbell Template Prep Kit (PacBio), following the manufacturer’s recommendations. The library was sequenced on the PacBio Sequel platform. The raw sequences were initially processed through the PacBio SMRT portal. The clean reads were obtained by comparing them with the reference database using the UCHIME algorithm (28) to detect chimera sequences, which were subsequently removed. The Uparse v7.0.1001 (29) was used to analyze sequences and annotate them with SSUrRNA Database (30) of Silva Database (31). The bacterial gut microbiota data underwent filtration based on relative abundance, excluding values below 0.01% and samples representing less than 10% at the phylum, genus, and species levels. Heatmaps were based on the pheatmap package in R (v4.2.1). Venn diagrams were based on the VennDiagram package.

### Statistical analysis

The data were expressed as the mean ± standard error of the mean (SEM). Statistical analysis was performed using ImageJ 1.46r, SPSS 26, R-studio, and GraphPad Prism 8 software. Group comparisons were assessed using the Student’s *t*-test, one-way analysis of variance, and Tukey’s multiple-comparison test. Statistical significance was denoted by asterisks (*P < 0.05, **P < 0.01, and ***P < 0.001). The correlation analysis of inflammatory factor and serum parameters was computed by Spearman.

## RESULT

### Probiotic characteristics of *B. halotolerans* SW207 *in vitro*

**Fig 2:**
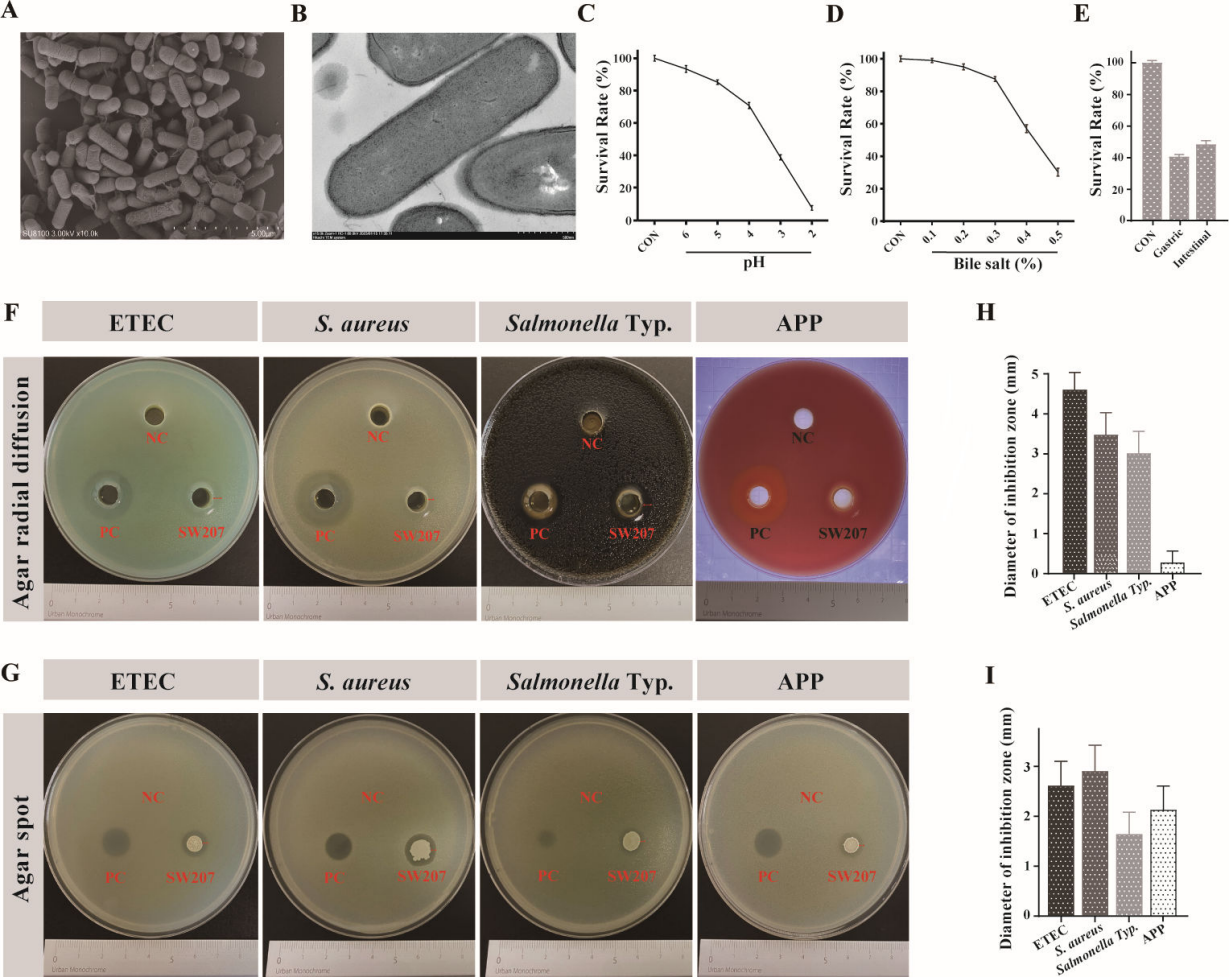
Colony morphology photos of SW207 by using (**A**) scanning electron microscope and (**B**) TEM, with the measurement scale under the figure. Survival rate of SW207 cells in (**C**) low pH, (**D**) bile, and (**E**) swine GIT fluids. Bactericidal activity of SW207 (**F**) CFS antibacterial and (**G**) agar spot antibacterial. (**H**) Diameter of inhibition zone analysis of CFS antibacterial. (**I**) Diameter of inhibition zone analysis of agar spot antibacterial. The data are presented as the mean ± SEM from a minimum of three distinct experiments.

Probiotics necessitate a requisite degree of resilience to successfully access the host organism and exert their influence. Within the scope of this investigation, we emulated the digestive milieu of various pH levels, concentrations of porcine bile salts, and the intricacies of porcine gastrointestinal fluids to determine the stress resistance of SW207. Results showed that the strain could survive at pH 3 and reached half of the survival rate at pH 4 but hardly live at pH 2 (Fig. 2C). SW207 can survive at all bile salt concentrations, and there was no difference in the survival rate between the CON group and the strain at 0.1% and 0.2% bile salt concentration (Fig. 2D). Upon reaching a concentration of 0.5%, the strain experienced a significant decline in survival rate, reducing to less than 50% of its initial value (Fig. 2D). In order to assess the viability of SW207 upon entry into the gastrointestinal tract, a co-cultivation experiment was conducted, wherein the strain was exposed to freshly harvested gastrointestinal collection fluids for a duration of 3 h. It was observed that, in comparison to the untreated control group, the impact of intestinal fluid was less pronounced than that of gastric fluid, yet both collectively maintained an overall survival rate of approximately 40%–50% (Fig. 2E). This suggests the potential for SW207 to establish residence and subsist within the gastrointestinal milieu.

We further employed the cell-free supernatant of SW207 to assess its bacteriostatic potential against ETEC and several other prevalent pathogenic strains. Following 24 h, the results unveiled SW207’s most robust bacteriostatic efficacy against ETEC (Fig. 2F), manifesting a substantial inhibition zone spanning 4–5 mm (Fig. 2H). Furthermore, its inhibitory reach extended to approximately 3 mm for *S. aureus* and *Salmonella* Typhimurium (Fig. 2H), underscoring SW207’s capacity to generate antimicrobial substances that directly impede pathogen growth. In an examination of SW207’s contact inhibition effect on these pathogens, they were cultivated on agar plates, whereupon conspicuous zones of inhibition were observed for all tested pathogens (Fig. 2G). Similarly, SW207 has the ability of direct contact inhibition to ETEC and several other pathogens.

### Evaluation of SW207 against bacterial diarrhea in piglets

**Fig 3:**
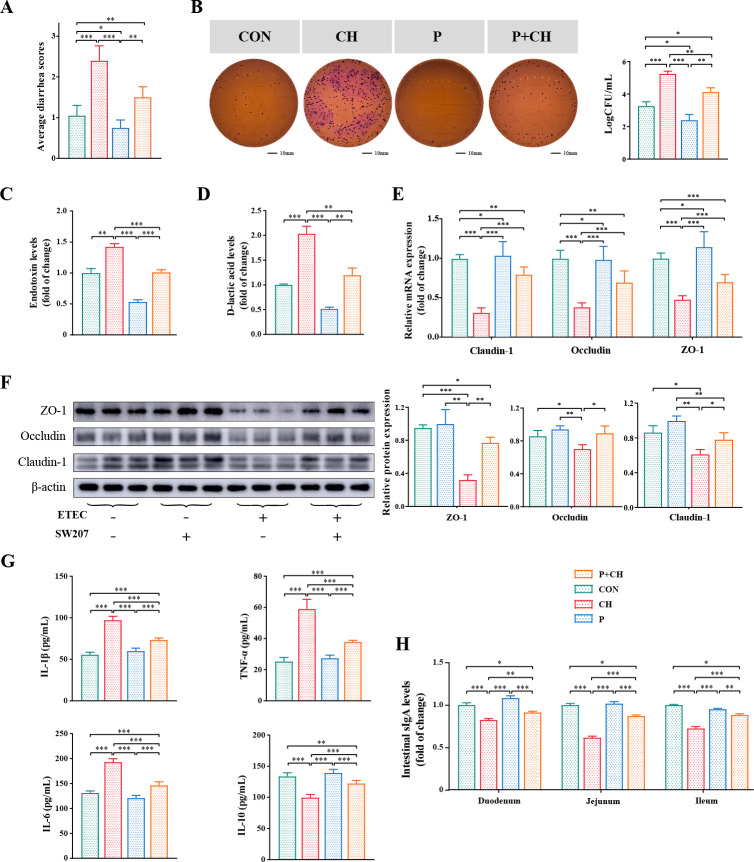
Survivability in GIT environment of SW207. (**A**) Average diarrhea scores. (**B**) Determination of *E. coli* load in cecum contents. (**C**) The serum endotoxin and (**D**) D-lactic acid levels of piglets. The impact of SW207 on the integrity and functionality of the ileal barrier. (**E**) Relative mRNA levels of tight junction genes. (**F**) Relative protein levels of tight junction. The impact of SW207 on serum oxidative stress, cytokine, and intestinal epithelial immunological barrier functions. (**G**) IL-1β, TNF-α, L-6, and IL-10 contents of serum. (**H**) The concentrations of secretory IgA in small intestinal. The data are presented as the mean ± SEM from a minimum of three distinct experiments. *P < 0.05; **P < 0.01; ***P < 0.001.

To confirm the antagonistic effect of SW207 on enterotoxigenic *E. coli*, we fed weaned piglets SW207 and conducted related experiments and evaluations (Fig. 1). The results of diarrhea scores show that compared with the CH group, the diarrhea rate of piglets in the CON group (*P* < 0.001), P group (*P* < 0.001), and P + CH group (*P* < 0.01) decreased significantly (Fig. 3A).

Furthermore, by quantifying the presence of *E. coli* in the cecal contents of different groups, we may also elucidate the probiotic’s capacity to counteract diarrheal episodes caused by this bacterium. Toward the end of the experiment, homogenization of cecal contents in the CH group yielded a large population of *E. coli* at 1.8 × 10^5^ CFU/mL (Fig. 3B). However, when pre-treated with SW207 as a preventive measure before the challenge, the *E. coli* count in the intestine significantly decreased to 1.5 × 10^4^ CFU/mL, demonstrating a notable reduction (*P* < 0.01, Fig. 3B). Although there was still some difference compared to the CON group, the piglets in the P group, which received probiotics alone, exhibited a significantly lower *E. coli* count in the intestine (3.0 × 10^2^ CFU/mL) compared to the CON group (2.0 × 10^3^ CFU/mL, *P* < 0.01, Fig. 3B). These outcomes underscore the potential of SW207 treatment in diminishing the incidence of diarrhea in weaned piglets.

Disease conditions often result in compromised intestinal barrier function, leading to an augmented translocation of endotoxins and various inflammatory mediators. Our results found that orally gavaged piglets with SW207 significantly reduced the levels of serum endotoxin (*P* < 0.001, Fig. 3C) and D-lactic acid (*P* < 0.01, Fig. 3D) upregulated by ETEC and reduced the damage of intestinal mucosal barrier. These findings suggest that SW207 enhances the intestinal epithelial barrier function in weaned piglet.

### Impact of *B. halotolerans* SW207 on tight junctions in the ileum

In order to explore the role of SW207 in intestinal epithelial barrier function, we detected the tight junction protein in the ileum of piglets as an evaluation of intestinal function. The findings indicate a significant reduction in the mRNA expression of ZO-1 (*P* < 0.001), occludin (*P* < 0.001), and claudin-1 (*P* < 0.001, Fig. 3E) in the P + CH group, which compared with the CH group. The protein expressions of ZO-1 (*P* < 0.01), occludin (*P* < 0.05), and claudin-1 (*P* < 0.05, Fig. 3F) in the P + CH group were also significantly higher than the CH group. The protein expression levels and mRNA levels of intestinal barrier function genes were consistent on the whole. Meanwhile, after orally gavaging SW207, the expression level of tight junction protein also had an increase (Fig. 3F). In summary, supplementation of SW207 contributes to the stability of the intestinal barrier function to a certain extent.

### *B. halotolerans* SW207 reduces ETEC-induced serum inflammatory cytokine expression and intestinal mucosal sIgA level

To assess the potential of SW207 in attenuating the inflammatory response and immunosuppression induced by ETEC, we performed an analysis of inflammatory cytokines in the serum. The serum pro-inflammatory cytokines IL-1β, TNF-α, IL-6, and IL-10 (Fig. 3G) in the P and CON groups had no marked difference (*P* > 0.05), but there was a significant difference in levels between the P + CH and the CH groups (*P* < 0.001); the P + CH group significantly reduced the upregulation of inflammatory factor levels caused by ETEC infection and significantly increased the IL-10 expression. In addition, although there were certain differences between the P + CH group and the CON group, it could be observed that the sIgA levels in the duodenum (*P* < 0.01, Fig. 3H), jejunum (*P* < 0.001), and ileum (*P* < 0.001) of the CH group were significantly increased by supplementing probiotics. We found that SW207 can partially inhibit and slow down the development of inflammation.

### *B. halotolerans* SW207 reduces ETEC-induced serum oxidative stress biomarkers

**Fig 4:**
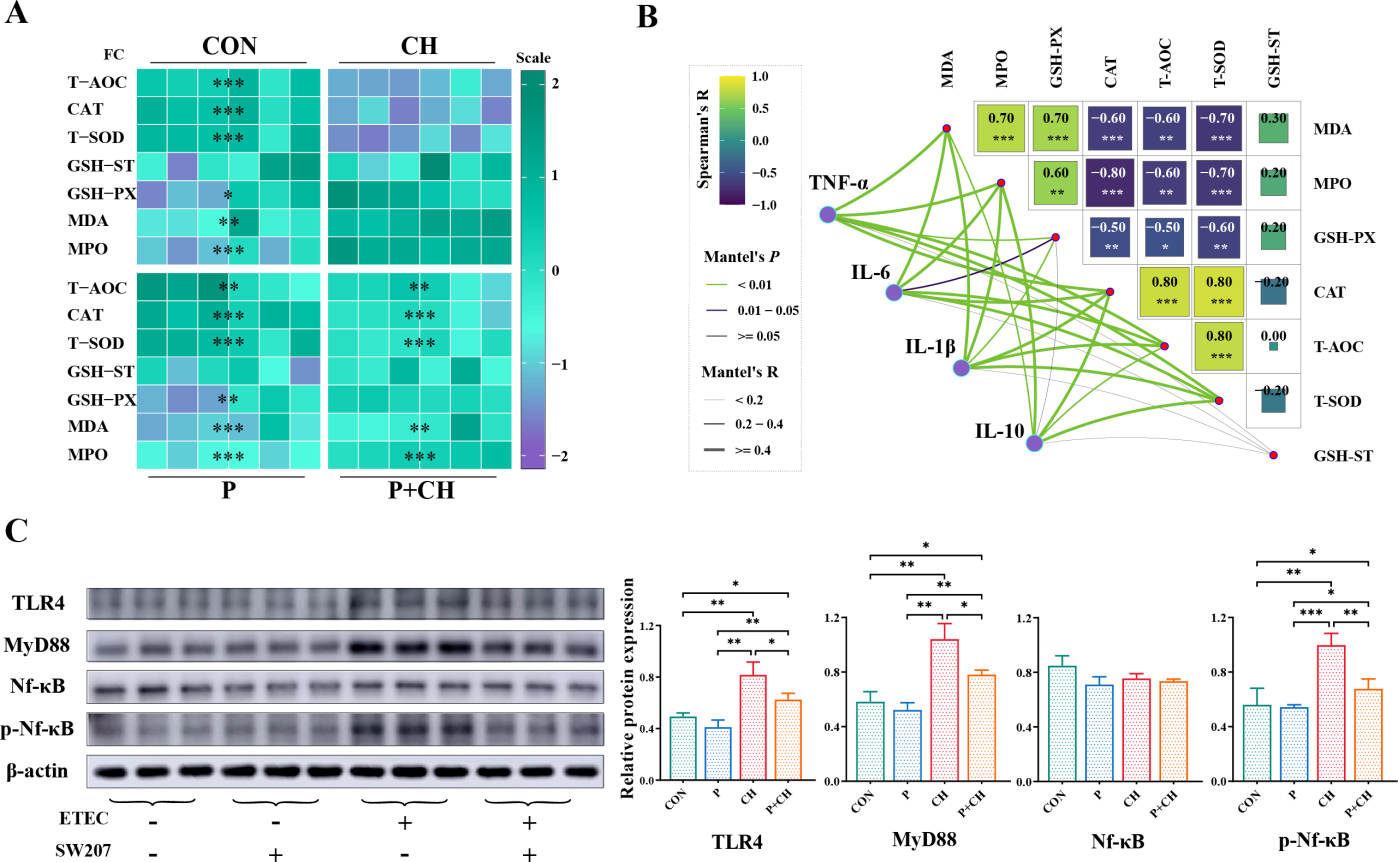
(**A**) Relative mRNA levels of serum oxidative stress responses (GSH-PX, T-AOC, T-SOD, GSH-ST, MDA, MPO, and CAT). Significance represents the result compared with the CH group. (**B**) Correlation analysis between serum oxidative stress responses and cytokine of relative mRNA levels. (**C**) The relative expression of TLR4/MyD88/NF-κB at the protein level. The data are presented as the mean ± SEM (*n* = 6). *P < 0.05; **P < 0.01; ***P < 0.001.

The detection of biomarkers for serum oxidative stress in weaned piglets after intervention with ETEC and SW207 is crucial information. As shown in the Fig. 4A, compared with the CH group, orally gavaged SW207 significantly upregulated the activities of CAT (*P* < 0.001), T-AOC (*P* < 0.001), and T-SOD (*P* < 0.001). At the same time, the levels of MDA (*P* < 0.01), GSH-PX (*P* < 0.05), and MPO (*P* < 0.001) were significantly reduced. However, there was no discernible variation in GSH-ST levels between the CH group and the remaining groups (*P* > 0.05, Fig. 4A). Through correlation analysis, the increase in IL-10 is not as significant as other genes, and the level of TNF-α has the greatest impact on other biomarkers of oxidative stress (Fig. 4B). In conclusion, the utilization of SW207 led to an augmentation in the levels of antioxidant defense system constituents, alleviating *in vivo* oxidative stress induced by pathogens and mitigating serum toxicity.

### The effects of *B. halotolerans* SW207 on the TLR4/MyD88/NF-κB signaling pathway in the ileum

The heat-stable toxin and heat-labile toxin produced by ETEC, along with Lipopolysaccharide (LPS), interact with host cells, triggering an inflammatory response in the immune system (1). TLR4 is a receptor that recognizes LPS and can activate immune and inflammatory responses, partly through both MyD88-dependent and MyD88-independent signaling pathways (14). These pathways lead to molecular signal cascades, ultimately promoting NF-κB nuclear translocation and the regulation of various inflammatory factors. At the translation level, we observed a consistent overall trend in the P group compared to the CON group, with a decrease that did not reach statistical significance (*P* > 0.05). However, when comparing the CH group and the P + CH group, there was a significant decrease in TLR4 (*P* < 0.05), MyD88 (*P* < 0.05), and phosphorylated NF-κB (*P* < 0.01).

### *B. halotolerans* SW207 enhances intestinal barrier against ETEC infection

**Fig 5:**
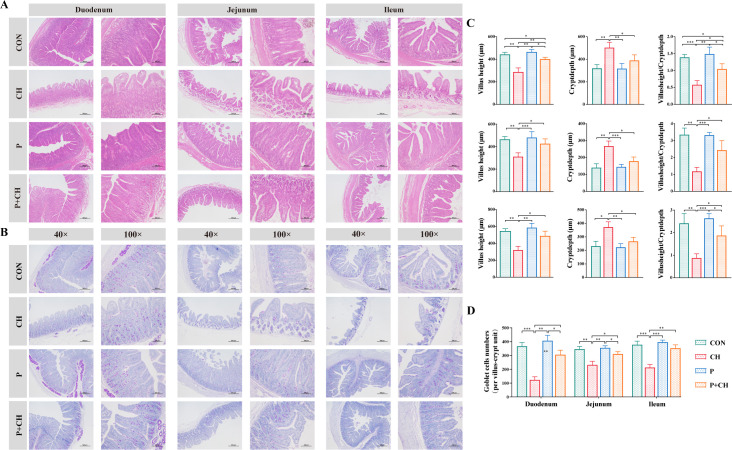
The impact of SW207 on gut barrier function. Intestinal morphology of (**A**) H&E and (**B**) PAS staining. (**C**) Statistical analysis of villus height, crypt depth, and villus height/crypt depth. (**D**) Statistical analysis of intestinal goblet cells numbers. The data are presented as the mean ± SEM from a minimum of three distinct experiments. *P < 0.05; **P < 0.01; ***P < 0.001.

The measurement of intestinal villi length and crypt depth can serve as indices for the evaluation of intestinal function. The duodenal villi in CON and P groups were finger shaped, and the villi and crypts were neatly arranged (Fig. 5). In contrast, the CH group exhibited limited duodenal recess, villi rupture, and hollowed villi, along with varying degrees of infiltration by inflammatory cells. The villus length and crypt depth in the P + CH group were significantly higher than the CH group (*P* < 0.05, Fig. 5C), and the villus length/crypt depth in the P + CH group was observably higher than the CH group (*P* < 0.05, Fig. 5C). The CH group exhibited pronounced inflammatory infiltration in the duodenal sections. It revealed that ETEC infection has a detrimental impact on intestinal development and function. However, this phenomenon was significantly changed, the length of small intestinal villi increased, and the depth of crypts became shallower after administration of SW207. Our findings indicated an improvement in intestinal mucosal integrity. The results of PAS staining in intestinal tissue showed that SW207 had no significant effect on the number of intestinal goblet cells (secreting mucin) of early weaned piglets (*P* > 0.05, Fig. 5B and D) but significantly decreased the number of intestinal goblet cells in the CH group.

### Effect of *B. halotolerans* SW207 on the intestinal microbes

**Fig 6:**
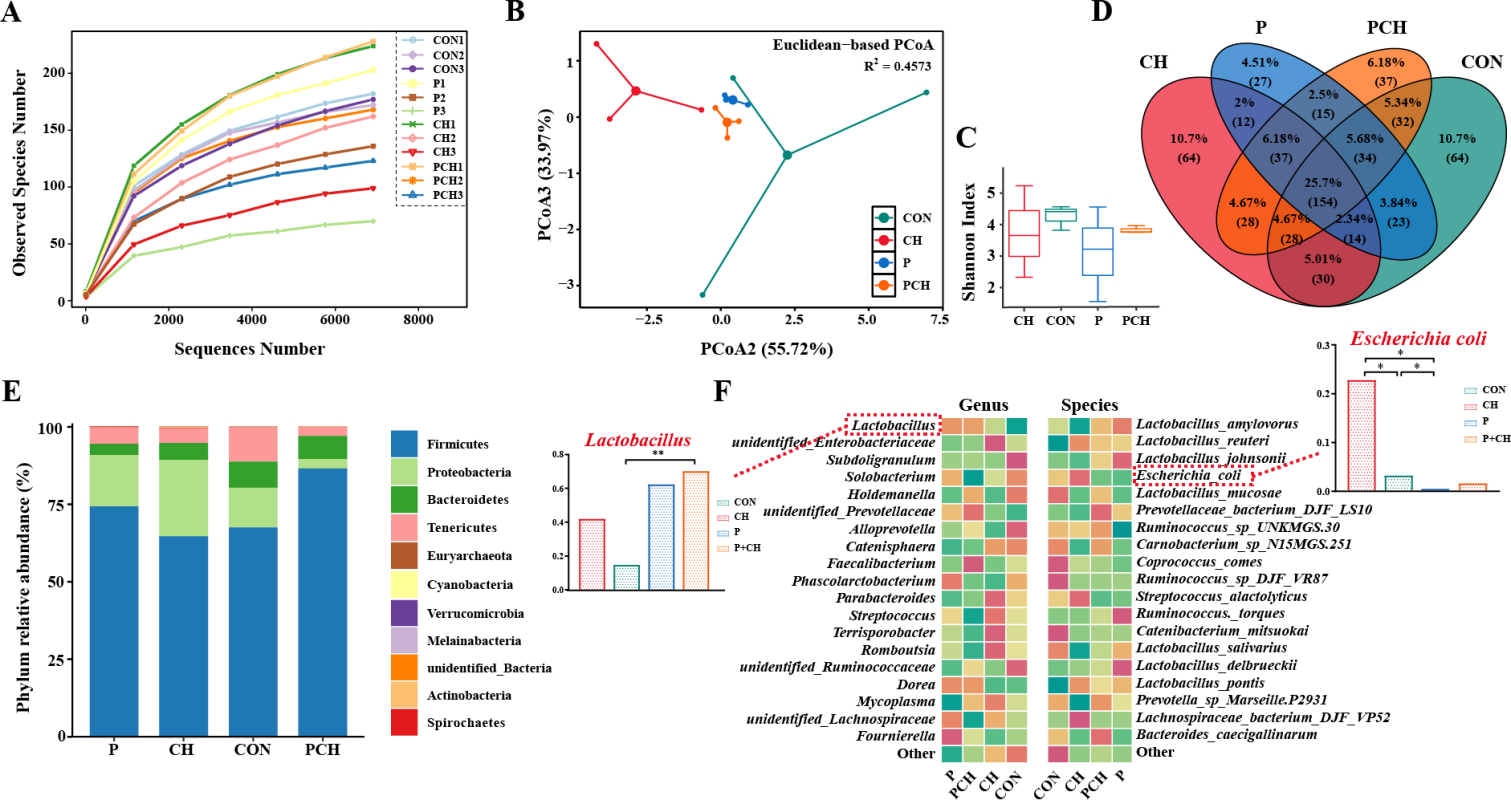
The impact of SW207 on cecal flora. (**A**) Observed specifications index, (**B**) Euclidean PCoA analysis index, (**C**) Shannon index, and (**D**) Venn diagrams for OTUs composition. The bacteria composition at the (**E**) phylum, (**F**) genus, and species level. The data are presented as the mean ± SEM. *P < 0.05; **P < 0.01; ***P < 0.001.

We next investigated the effects of SW207 on intestinal microbiota of healthy and ETEC-infected weaned piglets. The observed species curves revealed the detection of nearly all bacterial species in the cecum (Fig. 6A). A total of 599 core operational taxonomic units (OTUs) were identified (Fig. 6D). Principal coordinate analysis (PCoA) utilizing Euclidean distance revealed distinct separation between the CON and CH groups. This observation suggests a significant alteration in the composition of intestinal microbial communities following the administration of ETEC (Fig. 6B). After taking SW207, for delving deeper into the changes occurring in the intestinal flora structure following administration of SW207, we used Shannon index to reveal the α-diversity. Our findings revealed that there was no significant alteration in the α-diversity of the intestinal microbiota (Fig. 6C). However, the PCoA-Euclidean distance between the P + CH and P groups separated significantly, and the trend was more inclined toward the CON group (Fig. 6B).

We conducted a comprehensive investigation into the alterations in cecal flora by following the administration of SW207; a total of 11 phylum, 68 genus, and 61 species were identified (Fig. 6E and F). We found that the relative abundance of Firmicutes in cecal microbial community of piglets decreased, and the relative abundance of Proteobacteria increased after infection with ETEC, but this situation was improved with oral gavage of SW207 (Fig. 6E). In addition, SW207 significantly increased the content of *Lactobacillus* by comparing with the CH group, and most of *Lactobacillus* bacteria represented intestinal beneficial bacteria (*P* < 0.01, Fig. 6F). Most obviously, through high-resolution sequencing, we found that ETEC infection led to a significant increase in *E. coli* in the CH group, while SW207 significantly changed this phenomenon, significantly reduced the *E. coli* content in the P + CH group (*P* < 0.05, Fig. 6F) and P group (*P* < 0.05, Fig. 6F), and reached the level of the CON group.

## DISCUSSION

Weaned piglets are crucial in production but often face ETEC infections leading to diarrhea and health issues. Given the stringent constraints on antibiotic use in the current landscape, probiotics emerge as an imperative alternative for health management (32). We identified the probiotic strain SW207 with strong stress resistance, reducing diarrhea rate, improving host intestinal health, and improving the ability to resist ETEC infection. Our exploration of the interplay between SW207 and ETEC mechanisms furnishes a scientific foundation for the enhancement of weaned piglets health and resilience against diarrhea through probiotic intervention (33, 34).

*B. halotolerans* SW207 was isolated from the cecum of a healthy Lei-Xiang pig (Fig. 2A and B). Probiotic passes through the stomach into the intestines. It needs to tolerate acidic gastric juices (35) and the decomposition of some digestive enzymes (33, 35). SW207 has good tolerance and survival ability in the simulated *in vivo* environment and can survive under the influence of gastric acid and gastrointestinal juice. Additionally, bile salt in bile is discharged from the liver into the small intestine (36), requiring the strain to possess the ability to tolerate bile salt. SW207 is also well tolerant to bile salts, and these tests reveal that SW207 has the potential to survive and colonize in the intestinal tract. Probiotics also need to have a certain role of anti-pathogenic microorganisms. As previous studies of *Bacillus* have concluded (10, 16), members of the *Bacillus* can produce a variety of antibacterial substances, including peptides and lipopeptide antibiotics and bacteriocin (10), which have obvious antagonistic effects on a variety of pathogens (37). However, previous studies have been based on the effect of contact inhibition (10, 16); our research has uncovered that SW207 can exert a direct antibacterial effect on pathogenic microorganisms through CFS with the production of secondary metabolites. This revelation suggests that SW207 may competitively inhabit the intestinal environment, diminishing the growth and proliferation of ETEC within the intestinal tract, thus reducing the incidence and severity of diarrhea while maintaining intestinal health.

The measurement of intestinal villi length and crypt depth can serve as indices for the evaluation of intestinal function (38, 39). Reduced villi length hampers nutrient absorption and impairs digestion. Increased crypt depth suggests underdeveloped intestinal villi and compromised secretory function (40). In this study, we found that ETEC infection has adverse effects on intestinal development and function. However, this phenomenon was significantly changed, the length of small intestinal villi increased, and the depth of crypts became shallower after administration of SW207. In addition, although SW207 did not increase the number of intestinal goblet cells in healthy piglets (*P* > 0.05), it significantly increased the level of ETEC-infected piglets, which agrees with previous studies (41). The observed reversal of this condition upon administration of SW207 suggests a potential pivotal contribution to intestinal immunity. Disease conditions often result in compromised intestinal barrier function (42), leading to an augmented translocation of endotoxins and various inflammatory mediators (13). Interestingly, the administration of SW207 was found to markedly reduce serum endotoxin and D-lactic acid levels (*P* < 0.01), enhancing their resilience against damage induced by ETEC. Moreover, SW207 also significantly elevates the levels of intestinal mucosal sIgA, enhancing intestinal mucosal health and integrity, thereby improving overall gut health.

One fundamental role of the intestinal mucosa is to establish a protective barrier between the gastrointestinal contents and the underlying immune system (38, 41). In the context of foreign pathogen invasion, the tight junction proteins play a predominant role in sealing the barrier of the piglets’ ileum (14), and the decreased expression of ZO-1, claudin-1, and occludin is correlated with the dysfunction of ileum barrier and the increase of permeability (11, 14). In this study, SW207 significantly elevates the protein expression levels of ileum ZO-1 (*P* < 0.01), claudin-1 (*P* < 0.05), and occludin (*P* < 0.05, Fig. 3F) between the CH and CH + P groups. This suggests the potential modulation of these pivotal proteins’ expression and function, fostering tight connections among intestinal mucosal cells, diminishing intestinal permeability, preserving intestinal health, and reducing the occurrence of diarrhea, which aligns with prior probiotic research (17, 33).

Alterations in cytokine levels serve as an indirect measure of the inflammatory response and play a pivotal role in modulating the integrity of the barrier in the small intestine (5, 43). Therefore, in this study, downregulation of tight connection-associated proteins induced by ETEC infection may also contribute to inflammatory penetration in the body. The results indicate that ETEC significantly upregulates serum inflammatory cytokine levels, including IL-1β, TNF-α, and IL-6 (*P* < 0.001), whereas the administration of SW207 can markedly mitigate this upregulation (*P* < 0.001). SW207 may achieve this effect through competitive inhibition of ETEC growth and proliferation, thereby reducing the immune response triggered by ETEC and alleviating the systemic inflammatory reaction. We reached similar conclusions to previous studies (14, 17, 41, 44), which showed that SW207 can partially inhibit and slow down the development of inflammation.

Evidence suggests that ETEC causes excessive oxidative stress beyond what the body can repair by disrupting the intestinal barrier, leading to abnormal inflammatory responses (1). The detection of biomarkers for serum oxidative stress in weaned piglets after intervention with ETEC and SW207 is crucial information (45, 46). We observed that SW207 significantly ameliorates ETEC-induced oxidative stress, as it notably downregulates several oxidative stress markers, including CAT, T-AOC, and T-SOD (*P* < 0.001). Additionally, the levels of MDA (*P* < 0.01), GSH-PX (*P* < 0.05), and MPO (*P* < 0.001) were significantly reduced. MDA, MPO, and T-SOD are the main biomarkers to characterize the level of oxidative stress, which is the cause of the toxic effects of many pathogens (47, 48). SOD and CAT are integral components of the antioxidant defense system, serving as reliable indicators of antioxidant capacity (49). Moreover, correlation analysis shows that TNF-α has the strongest correlation with the level of serum oxidative stress, which is consistent with the Sánchez-Medina’s result (50). TNF-α can lead to severe inflammatory process and oxidative damage. This suggests that SW207 may enhance its antioxidant properties, reducing the harmful oxidative substances produced by ETEC and minimizing damage to cells and tissues.

In recent years, the role of *Bacillus* species in inflammation and immune regulation has been extensively documented. *Bacillus* species exhibit robust antibacterial and anti-inflammatory activities while maintaining the stability of the gut microbiota. For instance, a previous study demonstrated that *B. amyloliquefaciens* 40 regulates piglet performance, antioxidant capacity, immune status, and gut microbiota. *B. licheniformis* PF9 has been found to potentially reduce inflammation-related cytokines and significantly enhance the integrity of IPEC-J2 cells, possibly by blocking the NF-κB signaling pathway. Similarly, in this study, SW207 was found to maintain the normal function of the intestinal immune barrier by modulating the TLR4/MyD88/NF-κB pathway. It was confirmed that the protective mechanism of SW207 on intestinal barrier is related to the inhibition of NF-κB activation through TLR4/MyD88 axis. These findings suggest that SW207 can reduce inflammation levels, balance immune function, and contribute to the maintenance of intestinal mucosal integrity and health, thereby reducing the invasion of ETEC.

Infection of pathogens can lead to intestinal flora disorder, and the destruction of flora balance will lead to a series of harmful effects, which will eventually lead to diarrhea (1, 51, 52). Compared with the CH group, the β-diversity of intestinal flora in the P + CH group was closer to that in the CON group after administration of SW207. It has been suggested that the closeness of the diversity trend represents a more similar gut health status of the two groups of hosts (53, 54). Our findings exhibited a resemblance to the results reported by Wang et al. that the relative abundance of Proteobacteria phylum significantly increased after ETEC infection (51). SW207 significantly increased the abundance of *Lactobacillus* in the intestinal tract while notably decreasing the presence of coliforms in the cecum. These results were corroborated through cecal content dilution plating, yielding consistent findings. Studies have shown that intestinal dysbiosis in piglets with diarrhea is primarily distinguished by a reduction of *Lactobacillus* and an increase in *E. coli* (55). The abnormal increase of *E. coli* abundance may also lead to changes in host oxidative stress and immune level (3, 55). Our findings support this conclusion; we also observed that this dysbiosis can be significantly reversed through the supplementation of SW207. These findings showed that SW207 could inhibit the colonization and proliferation of ETEC and improve the damage of intestinal microbial barrier function in weaned piglets.

### Conclusions

This study elucidates the regulatory role of SW207 in mitigating ETEC-induced weaned piglet diarrhea. This beneficial effect is associated with SW207’s restoration of microbial balance, encompassing the promotion of symbiosis among beneficial bacteria, suppression of pathogen proliferation, and reduction of oxidative stress. Additionally, SW207 upregulates the expression of ileal tight junction proteins while reducing serum endotoxin and D-lactic acid levels, elevating small intestine mucosal sIgA levels, and preserving intestinal epithelial integrity. SW207 inhibits the activation of the TLR4/MyD88/NF-κB pathway and modulates key points in the inflammatory signaling pathways. In summary, SW207 enhances intestinal barrier function from the perspectives of mechanical, immune, and biological barriers, thereby improving gut microbiota homeostasis. The findings of this study furnish a scientific foundation for the utilization of SW207 as a preventive measure against diarrhea induced by ETEC in weaned piglets and for improvement of intestinal health.

## Data Availability

Data will be made available on request. The 16S rDNA sequencing raw data have been saved in the China National Center for Bioinformation, with the accession code PRJCA017983.

## SUPPLEMENTAL MATERIAL

Table S1 spectrum.03988-23-s0001.xlsx qPCR primers.

ASM does not own the copyrights to Supplemental Material that may be linked to, or accessed through, an article. The authors have granted ASM a non-exclusive, world-wide license to publish the Supplemental Material files. Please contact the corresponding author directly for reuse.
